# Evaluating a 5G smart first-aid care platform for streamlining prehospital care in acute cardio-cerebrovascular emergencies

**DOI:** 10.3389/fcvm.2026.1800890

**Published:** 2026-07-27

**Authors:** Zhihao Huo, Shaojuan Huang, Xuwei Liu, Zhengda Luo, Chunmei Liu, Qi Tang, Rong Li, Jian Luo, Dan Xie, Bisheng Shen, Jianyi Zhou

**Affiliations:** 1 The Eighth Clinical Medical College of Guangzhou University of Chinese Medicine, Foshan City, Guangdong Province, China; 2 Department of Emergency Medicine, Foshan Hospital of Traditional Chinese Medicine, Foshan City, Guangdong Province, China

**Keywords:** acute coronary syndrome, emergency medical services, ischemic stroke, prehospital emergency care, telemedicine

## Abstract

**Objectives:**

This study aims to evaluate the role of the 5G technology combined with intelligent medical treatment, 5G smart first-aid care platform in the prehospital emergency treatment of patients with acute ischemic cardio-cerebrovascular diseases.

**Methods:**

A single-center retrospective before-and-after study was conducted at Foshan Hospital of Traditional Chinese Medicine from January 2021 to June 2025. We evaluated its impact by comparing acute coronary syndrome (ACS) and acute ischemic stroke (AIS) patients transported by emergency medical services pre- and post-5G smart first aid care platform implementation.

**Results:**

A total of 367 cases were included in this study, with 180 in the controls group and 187 in the cases group. Implementation of the 5G smart first-aid care platform was associated with a significant reduction in first medical contact intervals times to chest pain/stroke team activation (*P* < 0.05); The generalized linear model analysis showed that 5G smart first-aid care platform was an independent factor associated with a shorter hospital stays[Exp(*B*) = 1.226, 95% CI: 1.002–1.501, *P* = 0.047].

**Conclusions:**

Implementing 5G smart first-aid care platform would optimize diagnostic efficiency, enhance the stability of the prehospital emergency system and shorten hospital stays.

## 1 Introduction

Cardio-cerebrovascular disease is the leading cause of death globally, with ischemic heart disease and stroke being the two most significant contributors, characterized by high prevalence, high disability rates, and high mortality (1). Its core treatment lies in restoring blood flow perfusion, reopening blocked vessels, and salvaging ischemic tissue within the shortest possible time. This process is highly time-dependent, with prompt intervention being critical to outcomes (2–4). Prehospital emergency care provides rescue services for patients from the onset of illness to their arrival at the hospital. In prehospital emergency care, shortening the “untreated period” plays an important role in improving the prognosis of patients (5). Traditional emergency medical services (EMS) communication, typically reliant on voice reports from the field to the emergency department (ED), creates bottle-necks. This fragmented information transmission delays diagnostic confirmation, defers specialist engagement, and compromises the preparation of in-hospital teams (6).

With the advancements in communication technology, telemedicine platforms have shown promising in bridging prehospital and in-hospital care (7, 8). The advent of fifth-generation (5G) wireless technology, with its advantages of enhanced broadband, multiple connections, and low latency, provides new opportunities for the development of prehospital emergency care. With the application of 5G technology since 2019 in China, the 5G smart medical prehospital first-aid model has been gradually put into practice in many big cities (9, 10). It enables the continuous and synchronous transmission of medical data from a moving ambulance directly to in-hospital. Such a 5G smart first-aid care plat-form was implemented in our hospital in 2023.

This study aims to retrospectively evaluate the impact of implementing the 5G smart first-aid care platform on key time-sensitive care metricss for patients with acute coronary syndrome and acute ischemic stroke, as well as hospital length of stay and mortality, and provide a basis for the promotion of such a 5G wireless technology-based medical management model.

## 2 Materials and methods

### 2.1 Study design and setting

A single-center, retrospective study was conducted at a large, urban and academic medical center. It is a tertiary (Grade A) public hospital in Guangdong Province, China, with 1,900 inpatient beds, has implemented an integrated patient management system spanning prehospital emergency response, in-hospital emergency care, and emergency intensive care. The ED, which was designated as a National Chest Pain Center and an Advanced Stroke Center, employs 110 medical staff, handles 120,000–130,000 emergency visits annually, and responds to over 6,000 emergency dispatches per year. The study period spanned from January 2021 to June 2025, encompassing the implementation of the 5G smart first-aid care platform, which was initiated in February 2023.

**Figure 1:**
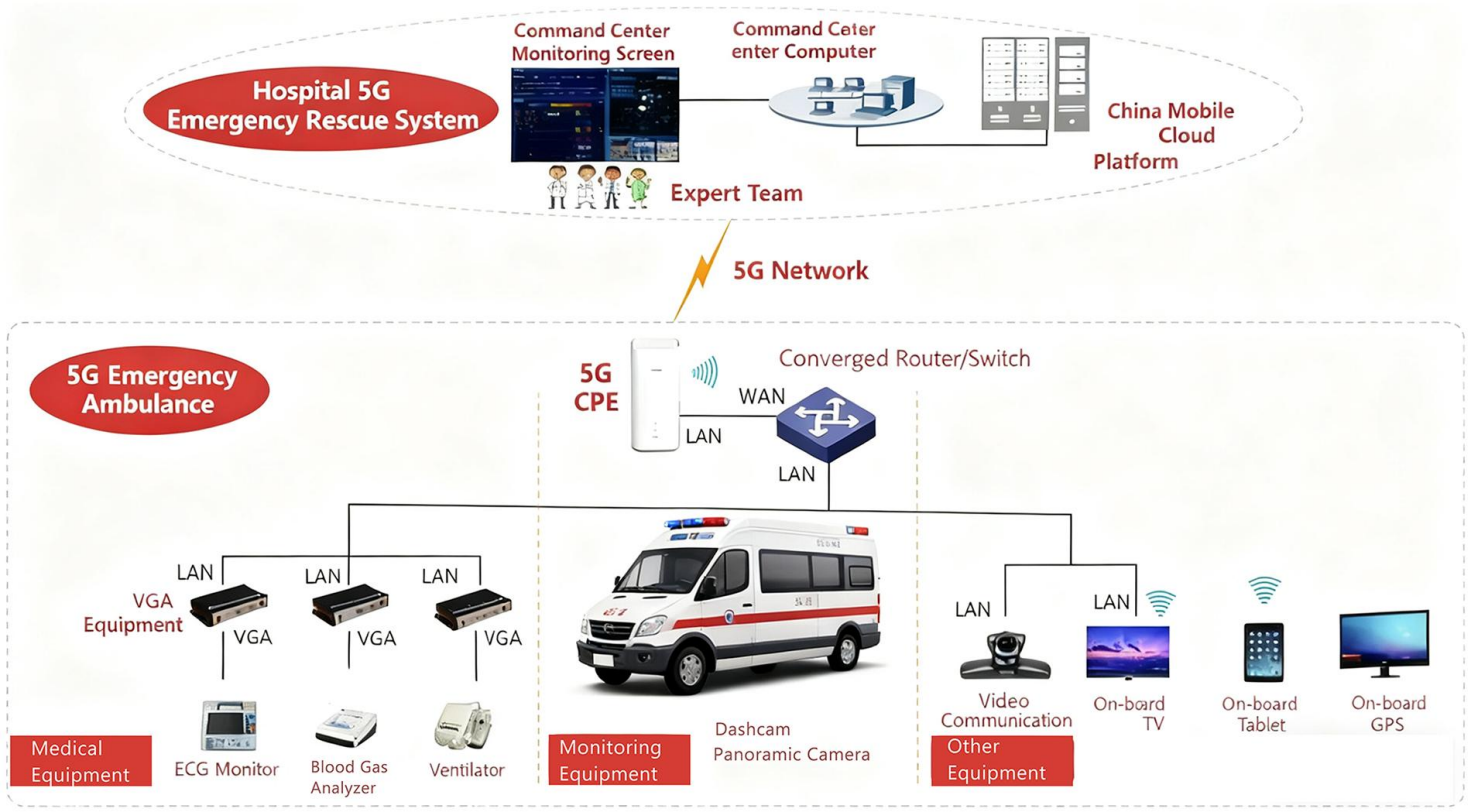
The composition of the 5G smart first-aid care platform.

The standard workflow of 5G smart first-aid care platform evaluated in this study have been described in detail by Tao Xiang in 2023 (10). In brief, the 5G-integrated emergency ambulance is equipped with mobile medical devices that connect directly to the 5G network via an onboard router and 5G CPE, establishing a link with the emergency response system to enable high-speed, reliable, and low-latency data transmission. The ambulance carries medical equipment such as electrocardiogram monitors, portable ECG devices, and blood gas analyzers, allowing real-time, lossless transmission of patients’ vital signs to the ED. This enables in-hospital physicians to assess patients’ conditions in advance and participate in prehospital care in real-time, establishing an intelligent emergency model characterized as “the hospital comes to the patients” once the patients enter the ambulance. The composition of the 5G smart first-aid care platform is illustrated in Figure 1. All emergency medical personnel involved are registered physicians or nurses who have received comprehensive training in both in-hospital and pre-hospital emergency care.

### 2.2 Prehospital emergency care

Patients in the controls group received conventional prehospital emergency care. After assessment at the scene, the patients were then transferred to the ambulance and transported to the hospital. Emergency medical personnel reported key findings by voice via phone, only to the emergency department doctor. Specialist teams were usually notified after the patient arrived and was assessed again in the emergency room.

Patients in the cases group were managed by applying 5G smart first-aid care plat-form on top of the controls group. From the moment care started in the ambulance, vital signs, ECG, and video were sent continuously and simultaneously. Patients with suspected ACS or AIS received real-time teleconsultation from the in-hospital chest pain center or stroke center team, enabling the timely preparation of subsequent rescue measures before hospital arrival.

### 2.3 Participants

**Figure 2:**
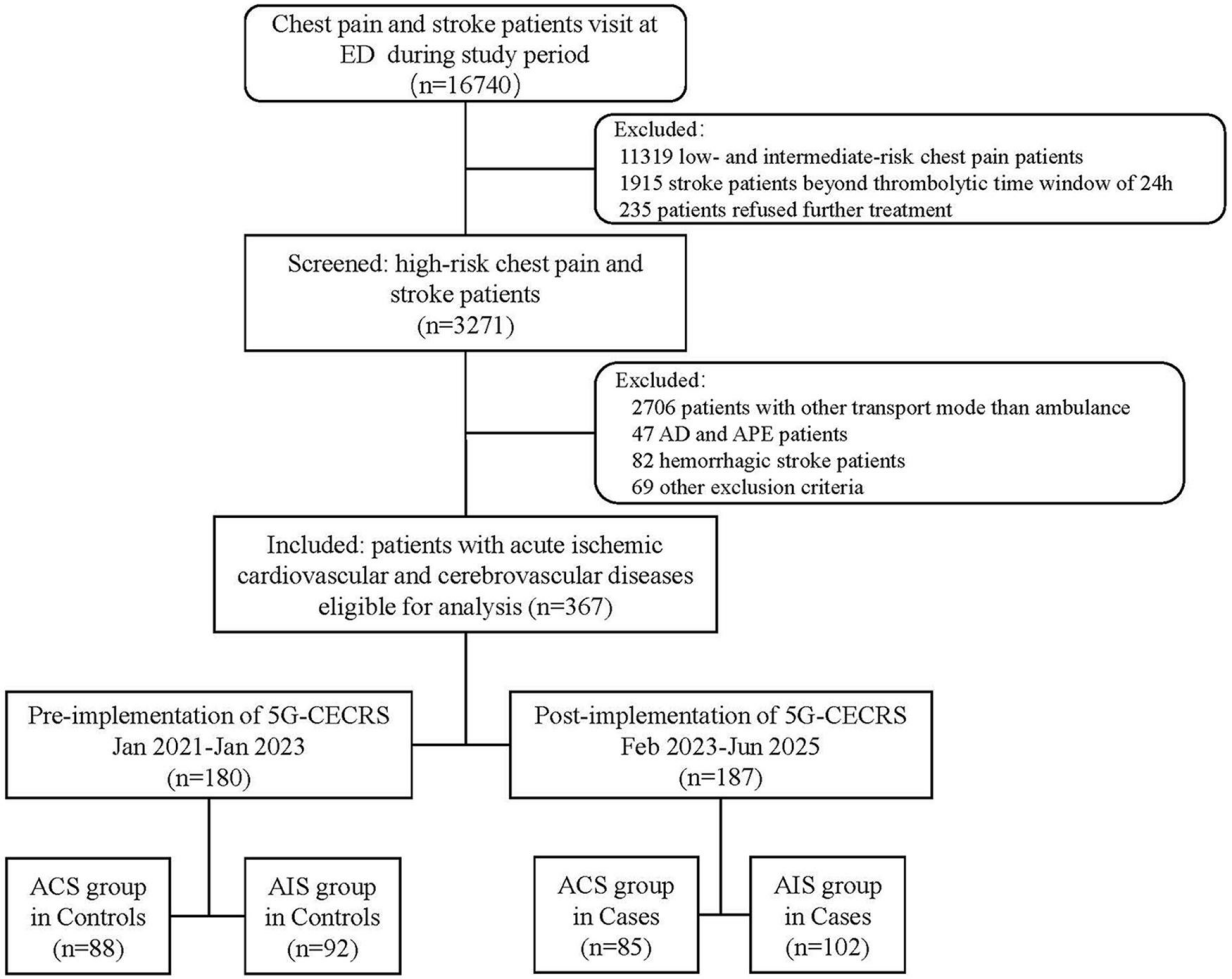
The flowchart of the inclusion process for study participants.

All retrospective patient data were retrieved from the electronic medical records (EMR) system. Patients with ACS or AIS who were admitted to the ED between January 2021 and June 2025 via EMS were included. The exclusion criteria were as follows: 1) had severe organic diseases or malignancies; 2) had a history of psychiatric disorders; 3) inability to cooperate with treatment; 4) interhospital transfer after initial admission; 5) unavailability of the catheterization laboratory or interventional procedure room; 6) missing key time points of emergency resuscitation or incomplete medical records. The flowchart of the inclusion process for study participants is shown in Figure 2.

### 2.4 Data collection and outcome measures

The study variables included baseline characteristics and clinical profiles, such as age, gender, and medical history. For patients with ACS, efficiency metrics of emergency care were evaluated, including dispatch-to-arrival-at-patient time, first medical contact intervals times to-electrocardiogram (FMC2ECG), to-chest pain team activation, and to-initial diagnosis. Besides, door-to-cardiac catheterization laboratory (D2C) time, and door-to-wire (D2W) time were also measured. For AIS patients, the following time-based indicators were assessed: dispatch-to-arrival-at-patient time, first medical contact inter-vals times to stoke team activation, door-to-needle time (DNT), door-to-puncture time (DPT). The primary outcome measures included mortality and length of hospital stay (LOS). All data were also extracted from the EMR system.

### 2.5 Statistical analysis

All statistical analyses were performed using SPSS. Normality and distribution of continuous variables were assessed using the Shapiro–Wilk test. The normally distributed data were presented as means and standard deviations (SD), whereas non-normally dis-tributed data were presented as medians and interquartile ranges (IQR). Categorical data were presented as frequency with percentage. The characteristics of the patients were compared using the Chi-square test for categorical data, as well as Student's *t*-test and Wilcoxon rank-sum test for the continuous data, respectively. To assess the independent effect of 5G smart first-aid care platform on the LOS, we constructed a generalized linear model (Gamma regression, log-link function) and adjusted for disease type, medical his-tory, gender and age. The results were presented as the adjusted rate ratio and its 95% confidence interval. For all analyses, *P* < 0.05 was considered significantly different. Missing data were excluded from the analysis.

### 2.6 Ethical approval

This study was approved by the institutional review board of Foshan Hospital of Traditional Chinese Medicine (approval number: KY[2023]264) and conformed to the provisions of the Declaration of Helsinki, as revised in Fortaleza, Brazil, in October 2013. The need for informed consent was waived owing to the retrospective nature of the study.

## 3 Results

There were 367 participants included, with 180 assigned to the controls group and 187 to the cases group. As shown in Table 1, no significant differences were observed in demographics, medical history, diagnosis, and interventions (*P* > 0.05). This indicates that baseline characteristics and disease severity were comparable.

**Table 1:** Patient demographics, medical history, diagnosis, and interventions of the total study population (*n* = 367).

Parameters	ACS		AIS		*P* ^a^	*P* ^b^
	Controls (*n* = 88)	Cases (*n* = 85)	Controls (*n* = 92)	Cases (*n* = 102)		
Gender, male, *n* (%)	56 (63.64)	65 (76.47)	50 (54.35)	59 (57.84)	0.066	0.624
Age, years, median (IQR)	73 (62–80.75)	66 (55–77)	75.5 (63.25–80)	70.5 (57.5–80)	0.061	0.071
Medical history, *n* (%)						
Diabetes	25 (27.71)	34 (39.56)	19 (20.65)	25 (24.51)	0.108	0.521
Hypertension	53 (60.23)	40 (47.06)	48 (52.17)	66 (64.71)	0.082	0.076
Hyperlipidemia	19 (21.59)	14 (16.47)	1 (1.09)	1 (0.98)	0.392	0.942
Prior cardiovascular disease	12 (13.64)	20 (23.53)	10 (10.87)	12 (11.76)	0.094	0.844
Diagnosis outcome					0.079	
UA, *n* (%)	16 (18.82)	9 (10.59)	—	—		
NSTEMI, *n* (%)	34 (38.65)	29 (34.12)	—	—		
STEMI, *n* (%)	38 (43.18)	47 (55.29)	—	—		
Interventions in ACS						
PCI, *n* (%)	33 (37.50)	44 (51.76)	—	—	0.059	
Interventions in AIS						0.372
IVT, *n* (%)	—	—	45 (48.91)	39 (38.24)		
EVT, *n* (%)	—	—	12 (13.04)	19 (18.63)		
Bridging therapy, *n* (%)	—	—	24 (26.09)	19 (18.63)		

ACS, acute coronary syndrome; AIS, acute ischemic stroke; IQR, interquartile ranges; UA, unstable angina; NSTEMI, non-ST-segment elevation myocardial infarction; STEMI, segment elevation myocardial infarction; PCI, percutaneous coronary intervention; IVT, intravenous thrombolysis; EVT, endovascular therapy.

a *P*: ACS-Controls compared with ACS-Cases.

b *P*: AIS-Controls compared with AIS-Cases.

Following the implementation of the 5G smart first-aid care platform, significant changes were observed in efficiency metrics for patients with ACS (Table 2). The notifica-tion process was expedited, with a median time of 1 (IQR: −2, 6) min from FMC to chest pain team activation. Time metrics from FMC to critical interventions demonstrated rapid ACS identification, with a median time of 10 (IQR: 4–36) min from FMC to initial diagnosis. But in a subgroup analysis of STEMI patients undergoing PCI, D2C time (*P* = 0.253) and D2W time (*P* = 0.344) showed no statistically significant differences between the two groups.

**Table 2:** Comparing the ACS management characteristics before and after 5G smart first-aid care platform.

Parameters	ACS-Controls (*n* = 88)	ACS-Cases (*n* = 85)	*P*
Total ACS patients			
Dispatch-to-arrival-at-patient time, min, median (IQR)	11.5 (10–15.75)	11 (9–14)	0.159
FMC intervals times to			
ECG, min, median (IQR)	6.5 (3–14.5)	4 (2–9.5)	0.049
chest pain team activation, min, median (IQR)	11 (5–23.5)	6 (4–15)	0.020
initial diagnosis, min, median (IQR)	24 (7–50)	10 (4–36)	0.009
Mortality, n (%)	9 (10.22)	10 (11.7)	0.747
Hospital LOS, day, median (IQR)	9 (6–13)	7 (5–10)	0.086
PCI-treated STEMI patients (*n* = 24 VS. 32)			
D2C time, min, median (IQR)	41 (28–62)	39 (16–54.25)	0.253
D2W time, min, median (IQR)	63 (47–90)	60 (37.25–84)	0.344

ACS, acute coronary syndrome; IQR, interquartile ranges; FMC, first medical contact; ECG, electrocardiogram; STEMI, segment elevation myocardial infarction; PCI, percutaneous coronary intervention; D2C, door-to-cardiac catheterization laboratory; D2W, door-to-wire; LOS, length of stay.

As for the time metrics for AIS management after 5G smart first-aid care platform implementation, it was summarized in Table 3. The FMC intervals times to stroke team activation time was decreased from 23(18–31.25) to 10 (IQR: 7–11) min (*P* < 0.0001). But in a subgroup analysis of AIS patients receiving reperfusion therapy, among those eligible for intravenous thrombolysis (IVT), the DNT increased from 29 (IQR: 20–48.5) min to 37 (IQR: 28–49) min (*P* = 0.077). But the proportion of patients with DNT < 60 min increased from 84.44% to 92.37%, DNT ≥ 60 min decreased from 15.56% to 7.69%. Though the observed reduction showed no statistically significant, it may represent a clinically meaningful improvement, suggesting a reduction in delayed thrombolysis. The DPT of those meeting endovascular therapy (EVT) conditions was performed in a mean time of 138 ± 46.99 min.

**Table 3:** Comparing the AIS management characteristics before and after 5G smart first-aid care platform.

Parameters	AIS-Controls (*n* = 92)	AIS- Cases (*n* = 102)	*P*
Dispatch-to-arrival-at-patient time, min, median (IQR)	11 (8–13)	10 (7–13)	0.314
FMC intervals times to stroke team activation, min, median (IQR)	23 (18–31.25)	10 (7–11)	<0.0001
DNT, min, median (IQR) (*n* = 45 vs. 39)	29 (20–48.5)	37 (28–49)	0.077
DNT distribution, *n* (%)			
<60 min	38 (84.44)	36 (92.31)	0.327
≤30 min	23 (51.11)	12 (30.77)	
30–44 min	9 (20)	15 (38.46)	
45–60 min	6 (13.33)	15 (38.46)	
≥60 min	7 (15.56)	3 (7.69)	
DPT, min, mean ± SD (*n* = 13 vs. 19)	137.5 ± 57.72	138.5 ± 46.99	0.417
Hospital LOS, day, median (IQR)	11 (7–18)	10 (6–14)	0.150
Mortality, *n* (%)	6 (6.52)	1(0.98)	0.054

AIS, acute ischemic stroke; IQR, interquartile ranges; DNT, door-to-needle time; DPT, door-to-puncture time; SD, standard deviation; LOS, length of stay.

Comparison of mortality and length of stay between the two groups is shown in Tables 2, 3. No statistically significant difference was observed in mortality among ACS patients (Controls-ACS: 10.22% vs. Cases-ACS: 11.7%; *P* = 0.747). For AIS patients, the mortality rate decreased from 6.52% in the Controls-AIS group to 0.98% in the Cases-AIS group, indicating a strong trend toward reduction, though this difference did not reach the predefined level of statistical significance (*P* = 0.054).

Regarding the hospital length of stay (LOS), implementation of the 5G smart first-aid care platform was associated with a reduction in the median hospital LOS for both ACS and AIS. Among ACS patients, it decreased from 9 (IQR: 6–13) days to 7 (IQR: 5–10) days. Similarly, for AIS patients, the median hospitalization duration was shortened from 11 (IQR: 7–18) days to 10 (IQR: 6–14) days. To further evaluate the overall benefits of 5G smart first-aid care platform, we conducted a generalized linear model analysis on the combined hospitalization time of ACS and AIS patients. The result showed (Table 4) that the application of 5G smart first-aid care platform was an independent factor associated with a shorter hospital LOS [Exp(*B*) = 1.226, 95% CI: 1.002–1.501, *P* = 0.047]. Specifically, the hospital LOS for patients in the controls group was 22.6% longer than that in the 5G smart first-aid care platform group. Additionally, disease type itself had a significant impact on the LOS [ACS vs. AIS, Exp(*B*) = 0.782; 95% CI: 0.622–0.984; *P* = 0.036]. Moreover, the interaction between group and disease type was not significant (*P* = 0.752), indicating that the benefit of 5G smart first-aid care platform in shortening the hospital LOS was consistent across the two disease types. None of the other covariates in the model showed a significant effect.

**Table 4:** Generalized linear models analysis of factors related to LOS.

Factors	Exp(*B*)	95%*CI*	*P*
Group (Controls vs. Cases)	1.226	1.002–1.501	0.047
Disease type (ACS vs. AIS)	0.782	0.622–0.984	0.036
Diabetes (No vs. Yes)	0.903	0.752–1.084	0.275
Hypertension (No vs. Yes)	1.000	0.843–1.187	0.998
Prior cardiovascular disease (No vs. Yes)	1.036	0.818–1.313	0.769
Gender (Female vs. Male)	0.984	0.831–1.165	0.851
Age (per 1-year increase)	1.005	0.999–1.011	0.135
Interaction: Group × Disease type	0.949	0.686–1.313	0.752

## 4 Discussion

The implementation of 5G smart first-aid care platform was associated with opti-mized rescue processes, a reduction in extreme treatment delays, a promising trend in mortality risk reduction, and shortened hospital stays, underscoring its value for public health systems.

The first assumption was that the 5G smart first-aid care platform would enhance overall diagnostic efficiency. This was supported by a statistically significant reduction in both the FMC to chest pain team and stroke team activation. A shorter interval from symptom onset to treatment is consistently associated with greater patient benefit from reperfusion therapy (11). Delays in ECG acquisition have been linked to postponed reper-fusion, a higher incidence of major adverse cardiac events, and poorer long-term cardio-vascular prognosis (12). After the application of the 5G smart first-aid care platform, the median FMC2ECG time was 4 min, which was substantially below the sub-10-min benchmark recommended by the 2025 ACC/AHA Guidelines for the management of pa-tients with ACS (13). The third quartile was also reduced from 14.5 min to 9.5 min. These enhancements were mainly attributed to the high-speed, reliable and low-latency data transmission, which facilitated earlier clinical decision-making (14).

To further assess whether these gains in diagnostic efficiency translated into im-proved treatment timelines, we then focused on in-hospital management for STEMI pa-tients—a time-sensitive subgroup of ACS. However, no statistically significant differences were observed in in either the D2C time or the D2W time for patients undergoing PCI treatment. The median D2W time decreased from 63 min to 60 min. This result aligns with a Chinese multi-center analysis by Kong RR et al., which reported a median D2W time of patients with STEMI receiving PCI of 60 (IQR: 45–79) min following China Chest Pain Center accreditation in 16 tertiary Grade A hospitals, a figure also comparable to U.S. benchmarks (15, 16). Furthermore, the 2024 National Quality Control Report of Chest Pain Centers in China indicated an average D2W time (standard version) of 67.5 min nationwide and 61.1 min in Guangdong Province for STEMI patients receiv-ing PPCI (17). However, a 2020-year study by the German Chest Pain Center showed a notably lower median D2B time of 40 (IQR: 19–80) min during the 2008–2014 period (18). It can be seen that our cohort's performance is basically consistent with the national landscape in China, but there is still a certain gap compared to healthcare systems in well-resourced developed countries. This indicates that further measures need to be taken to optimize in-hospital workflows to shorten the core reperfusion time.

The second assumption was that the 5G smart first-aid care platform would enhance the stability of the pre-hospital emergency system. After its implementation, the median DNT for AIS patients eligible for IVT increased from 29 min to 37 min. Although the median DNT increased after the implementation of the 5G smart first-aid care plat-form, it still met the target range of DNT recommended by the international stroke guide-lines for Stroke Centers: for AIS patients receiving IVT, the basic target is to have 50% of patients within 60 min, with an advanced target of <45 min (19, 20). Data from The Third China National Stroke Registry (CNSR-III) showed that the median DNT for IVT patients was 60 min from 2015 to 2018, with 53.4% of patients having a DNT < 60 min and 33.1% having a DNT < 45 min. By 2019–2020, the median DNT at China Stroke Centers had improved to 45 min, with 75.43% of patients treated within 60 min (21). Data from the Target: Stroke study in the United States showed that at the end of the Phase II project (2014–2018), 75.4% of patients achieved the 60-min DTN tar-get and 51.7% achieved the 45-min DNT target, respectively (22). In our study, the pro-portion of IVT-treated patients with DNT < 60 min increased from 84.44% to 92.31% after 5G smart first-aid care platform implementation, though the rate of DNT < 45 min experienced a slight decline from 71.11% to 69.23%. This pattern suggests that the role of the 5G smart first-aid care platform may be more in reducing extreme delays and enhancing the stability of the emergency system, which means that a broader population benefit from timely IVT.

Despite these improvements, the average DPT for AIS patients eligible for EVT showed no significant change, remaining at 138.5 min, far exceeding the global 90-min standard. This finding is consistent with a single-center retrospective study from Thailand, where direct pre-hospital neurologist consultation and real-time remote supervision achieved a DPT of 135.57 ± 22.43 min, leading the authors to conclude that pre-hospital notification alone does not significantly shorten DPT and DNT (7). It also corresponds to our cohort's result that the D2W time for PCI treatment did not significantly improve, indicating that while prehospital and ED workflows were optimized, establishing an efficient response mechanism for the endovascular treatment team is a necessary measure to improve the quality of endovascular treatment (23).

The third assumption was that the 5G smart first-aid care platform would shorten the hospital LOS for both ACS and AIS patients. The hospital LOS of patients in the cases group were significantly shorter by approximately 18.4% (equivalent to 1–2 days) compared to the controls group. The observed reduction in hospital LOS also supports the notion that the 5G smart first-aid care platform facilitated more rapid diagnosis and treatment, which prevented further damage to the heart muscle/brain tissue, thereby reducing complications and accelerating clinical stabilization. In China, the median hospitalization duration of acute ischemic stroke patients is 11 days (24), and our results are basically consistent with this. However, a single-center study involving 703 patients in the United States showed that the median hospitalization duration of AIS patients treated with mechanical thrombectomy in this hospital was 6 (4–10) days (25), which may be related to the well-developed healthcare system in the United States. While the 5G smart first-aid care platform has an effect on shortening the hospitalization duration, measures such as implementing clinical pathway management and strengthening cooperation among medical alliance units should also be implemented to optimize patient prognosis and hospital re-source utilization.

Previous studies have confirmed that initiating the catheterization room before arrival at the hospital results in a significantly lower long-term mortality rate for STEMI patients, and the short-term mortality rate also tends to decrease, with statistically significant differences (16, 26, 27). Although the application of the 5G smart first-aid care platform significantly shortened the hospital LOS, it did not significantly improve the mortality rate in the study. Due to the extremely low number of death cases in this study (*n* = 26), our ability to conduct a reliable multivariate analysis was limited. Therefore, the conclusion regarding the impact of 5G smart first-aid care platform on the mortality rate of AIS patients should be regarded as preliminary and requires further verification in larger sample size studies.

## 5 Limitations

A primary limitation of this study lies in its observational design. Although we made rigorous efforts to adjust for known confounders, we cannot entirely rule out the potential influence of unmeasured confounding factors, such as the increasing experience of physicians over time, on the outcomes. As a single-center retrospective study, although the treatment process was standardized like that of the Advanced Stroke Centers and Chest Pain Centers, the generalizability of the research conclusion still needs to be further verified by future multi-center prospective studies. Regarding outcome measures, this study primarily focused on LOS, without follow-up evaluation of long-term neurological recovery, quality of life, or cost-effectiveness of the technology, all of which are critical for a comprehensive assessment of the value of a new healthcare intervention.

## 6 Conclusion

This single-center retrospective study demonstrates that implementation of the 5G smart first-aid care platform would optimize diagnostic efficiency, enhance the stability of the prehospital emergency system and shorten hospital stays. Furthermore, some targeted policies should also be developed to streamline the response of the endovascular therapy team in the hospital.

## Data Availability

The raw data supporting the conclusions of this article will be made available by the authors, without undue reservation.
